# Supplementary figures and images for: Role of YAP in early ectodermal specification and a Huntington's Disease model of human neurulation
Source: eLife. 2022 Apr 22;11:e73075. doi: 10.7554/eLife.73075 (PMC9033270; doi:10.7554/eLife.73075)

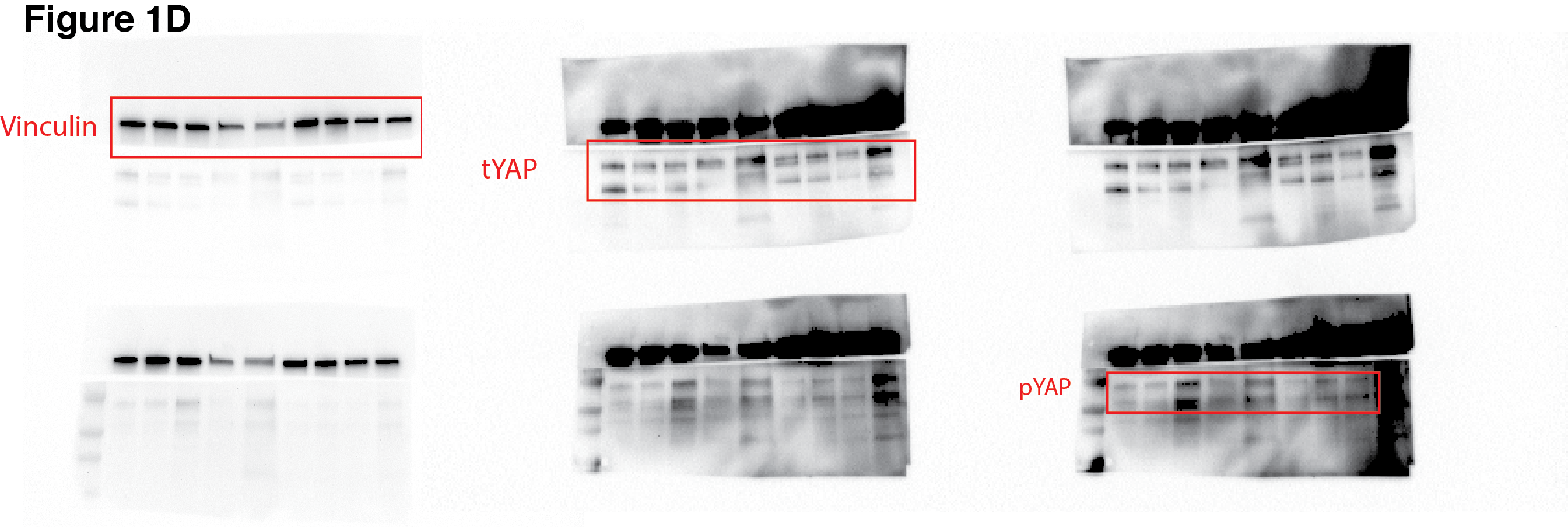

Supplement: Figure 1—source data 1. [file elife-73075-fig1-data1.zip › Figure1_source data1.png]

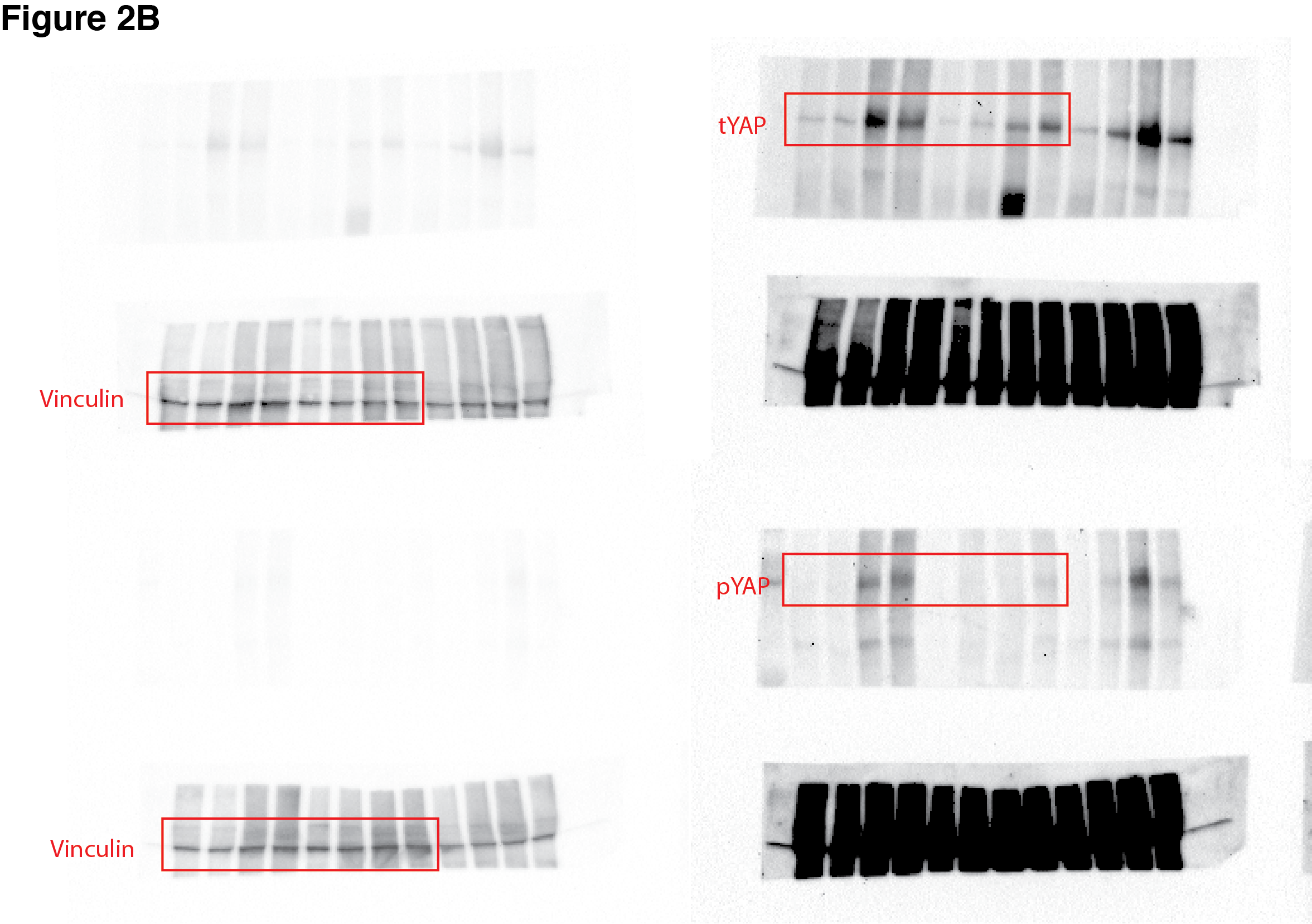

Supplement: Figure 2—source data 1. [file elife-73075-fig2-data1.zip › Figure2_source data1.png]

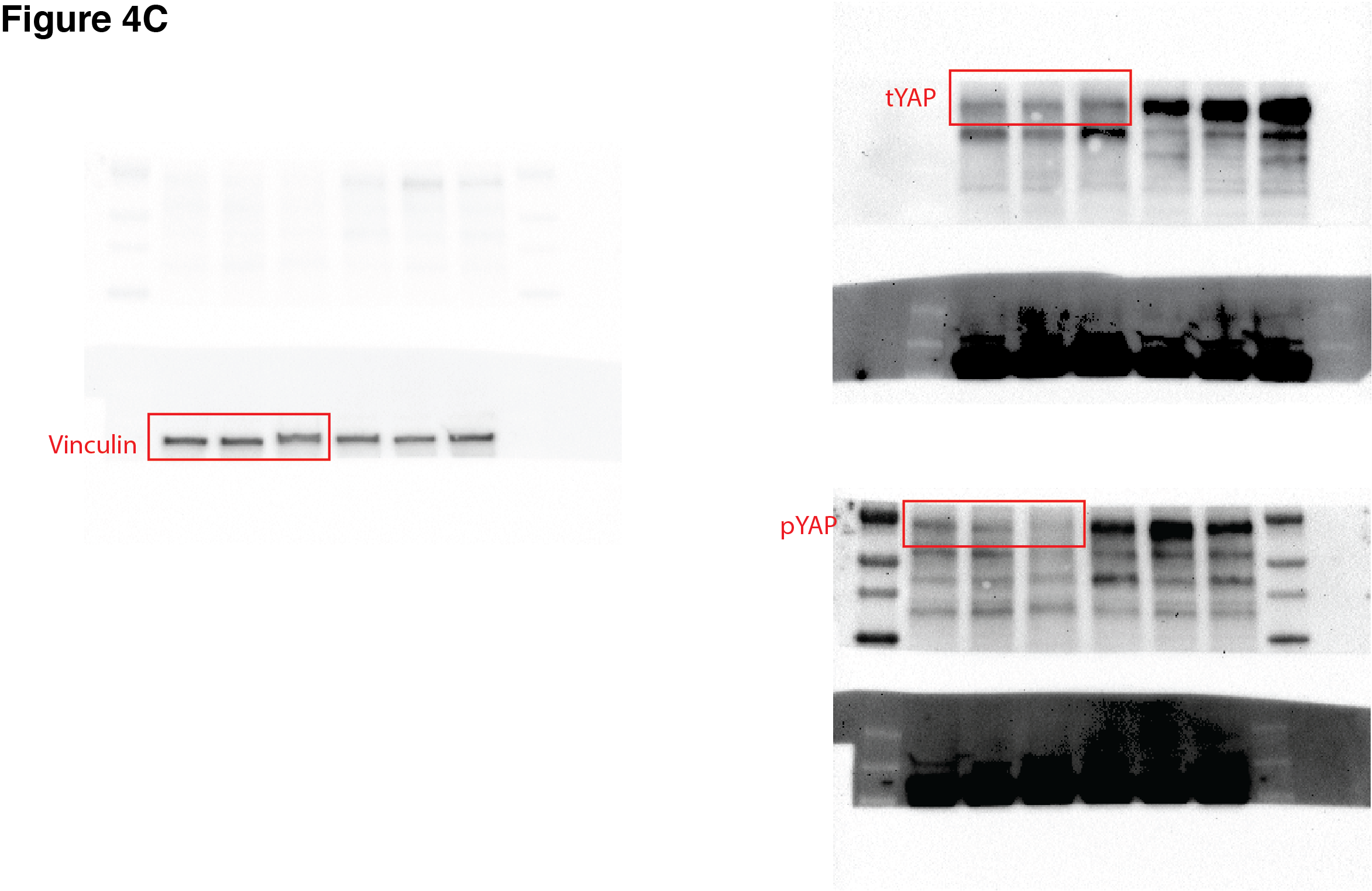

Supplement: Figure 4—source data 1. [file elife-73075-fig4-data1.zip › Figure4_source data1.png]
